# Where do obesity and male infertility collide?

**DOI:** 10.1186/s12920-024-01897-5

**Published:** 2024-05-10

**Authors:** Melika Jahangir, Majid Nazari, Emad Babakhanzadeh, Saeed Dehghan Manshadi

**Affiliations:** 1 Department of Pharmacy, Tehran University of Medical Sciences, P.O. Box: 64155-65117, Tehran, Iran; 2 Department of Medical Genetics, Shahid Sadoughi University of Medical Sciences, Yazd, Iran; 3 University of Science and Arts of Yazd, Yazd, Iran

**Keywords:** Male infertility, Obesity, Apolipoprotein B, Insilico, Gene expression, ARMS-PCR

## Abstract

The parallel rise in obesity and male infertility in modern societies necessitates the identification of susceptibility genes underlying these interconnected health issues. In our study, we conducted a comprehensive search in the OMIM database to identify genes commonly associated with male infertility and obesity. Subsequently, we performed an insilico analysis using the REVEL algorithm to detect pathogenic single nucleotide polymorphisms (SNPs) in the coding region of these candidate genes. To validate our findings in vivo, we conducted a comprehensive analysis of SNPs and gene expression of candidate genes in 200 obese infertile subjects and 240 obese fertile individuals using ARMS-PCR. Additionally, we analyzed 20 fertile and 22 infertile obese individuals using Realtime-qPCR. By removing duplicated queries, we obtained 197 obesity-related genes and 102 male infertility-related genes from the OMIM database. Interestingly, the *APOB* gene was found in common between the two datasets. REVEL identified the rs13306194 variant as potentially pathogenic with a calculated score of 0.524. The study identified a significant association between the AA (P value = 0.001) genotype and A allele (P value = 0.003) of the APOB rs13306194 variant and infertility in obese men. *APOB* expression levels were significantly lower in obese infertile men compared to obese fertile controls (*p* < 0.01). Moreover, the AA genotype of rs13306194 APOB was associated with a significant decrease in *APOB* gene expression in obese infertile men (*p* = 0.05). There is a significant association between the Waist-to-Hip Ratio (WHR) and LH with infertility in the obese infertile group. These results are likely to contribute to a better understanding of the causes of male infertility and its association with obesity.

**Supplementary Information:**

The online version contains supplementary material available at 10.1186/s12920-024-01897-5.

## Introduction

Infertility remains a significant global concern, affecting approximately 10–15% of couples worldwide, where male factors contribute to almost half of the cases [1]. Male infertility and obesity are interconnected health issues with potential associations [2]. The prevalence of obesity and infertility has simultaneously increased in modern societies, leading researchers to investigate their relationship [3]. Studies have revealed that an elevated body mass index (BMI) can negatively impact sperm quality and decrease the chances of fertility [4]. Obesity exacerbates infertility through various mechanisms. Metabolic dysregulation caused by increased adipose tissue affects testosterone secretion levels necessary for spermatogenesis [5]. Mitochondrial dysfunction and oxidative stress in sperm lead to DNA damage and apoptosis which is associated with reduced pregnancy rates [6, 7]. Epigenetic alterations, such as decreased methylation, have been observed in obese individuals and can affect sperm DNA [8]. Moreover, Genetic syndromes like Prader-Willi and Alstrom [9], which are characterized by obesity and infertility, further support the link between these conditions. Understanding the complex interplay between obesity and male infertility is crucial for effective management and treatment.

Numerous primary studies have employed various statistical methods to investigate the shared gene loci among different diseases [10]. Hu and Agarwal were the first to utilize expression data from different diseases to uncover their interrelationships [11]. Suthram et al. further expanded this approach by integrating protein network data from diverse diseases [12]. Linghu et al. developed a functional linkage network by merging 16 genomic characteristics, identifying potential associations between 110 diseases [13]. Common gene loci have been successfully identified across multiple diseases. Ellinghaus et al. performed a meta-analysis of GWAS studies to elucidate the genetic overlap between endometriosis and leiomyoma [14]. Adlam et al. employed LD score regression on the GWAS catalog database to delineate shared genomic loci between fibromuscular dysplasia and various conditions such as blood pressure, migraine, intracranial aneurysm, and coronary artery disease [15]. Furthermore, a study confirmed the presence of shared gene loci between body mass index and psychological diseases [16].

Although there have been numerous studies investigating the genetic associations of obesity with various diseases, there has been a notable absence of research exploring the shared genetic loci between obesity and male infertility [17]. Investigating the shared genetic factors and molecular pathways between obesity and infertility is crucial for a comprehensive understanding of their relationship and potential clinical implications. Through computational analyses, insilico experiments, and genotype analysis, we aim to unravel the underlying mechanisms NOA and obesity.

## Materials and methods

### Overlap between obesity and male infertility

Genes associated with male infertility and obesity were identified through a thorough search of the Online Mendelian Inheritance in Man (OMIM) database (omim.org). Specifically, the Gene Map Table within the database was utilized, which offers organized information on the relationships between phenotypes and genes. Duplicates were carefully eliminated to ensure accuracy in identifying the shared genes between male infertility and obesity. To further investigate these shared genes, the InteractiVenn tool (interactivenn.net) was employed, aiding in the visualization and analysis of the intersection between the two conditions.

### Protein-protein network and 3D structure

We used STRING databases (string-db.org) to identify protein-protein interactions between candidate genes for obesity and male infertility. The three-dimensional structure of APOB protein with and without rs13306194 polymorphism has been investigated by HOPE server (cmbi.ru.nl/hope).

### Insilico analysis of nsSNPs

**Fig. 1:**
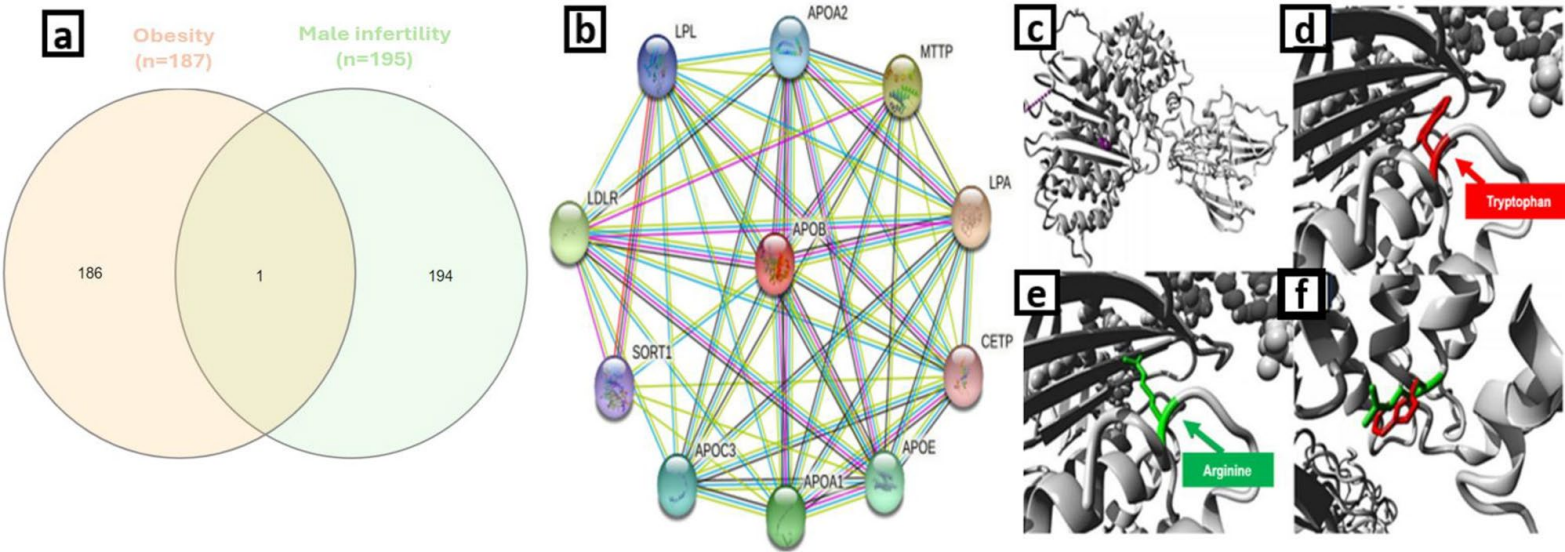
The tool HOPE was used to generate schematic representations of the original and mutant forms of APOB, specifically the Arg532Trp variant. The backbone of each amino acid is colored red, while the unique side chain is colored black. Homology models were created to illustrate the structural effects of the mutation. (**a**) The protein is shown in gray, and the mutated residue is represented by magenta spheres. Close-up images show the mutation with the mutated residue in red (**b**), the wild-type residue in green (**c**), and with both wild-type and mutated residues (**d**, **e**). (**f**) A representation of the gene-gene interaction of APOB as proposed by STRING is shown

SNPs of the candidate genes with a minor allele frequency (MAF) above 0.05 were obtained in FASTA format from the dbSNP database (ncbi.nlm.nih.gov/snp). The overall methodology employed in this study is visually represented in Fig. 1. The filtering process for the obtained SNPs relied on the Rare Exome Variant Ensemble Learner (REVEL) ensemble score [18], which has demonstrated superior accuracy in predicting the pathogenicity of non-synonymous SNPs (nsSNPs). Specifically, the REVEL algorithm incorporated 13 prediction scores encompassing function prediction scores (Sorting Intolerant from Tolerant or SIFT (blocks.fhcrc.org/sift), Polymorphism Phenotyping Version 2 or PolyPhen-2 (genetics.bwh.harvard.edu/pph2), Protein Variation Effect Analyzer or PROVEAN (jcvi.org/research/provean), likelihood ratio test or LRT, Variant Effect Scoring Tool or VEST (karchinlab.org/apps/appVest), Mutation Assessor (mutationassessor.org), Mutation Taster (mutationtaster.org), MutPred (mutpred.mutdb.org), FATHMM (fathmm.biocompute.org.uk/)) and conservation scores (SiPhy [19], PhyloP [20], GERP [21], phastCons [20]).

### Patients and biochemical tests

Between March 2017 and December 2020, 440 men were recruited for genetic testing. The study included 200 obese (BMI > 25 kg/m 2) infertile men and 240 obese fertile men. Eligible participants did not have serious endocrine or cardiovascular problems, alcoholism, drug addiction, psychiatric disorders, or recent participation (within the past six months) in weight loss programs. Body height was measured to the nearest centimeter with a rigid stadiometer. Weight, on the other hand, was measured to the nearest 0.1 kg using a calibrated scale while subjects were minimally clothed. To determine BMI, weight in kilograms was divided by the square of height. All cases of infertility were due to non-obstructive azoospermia (NOA), a condition in which abnormal sperm production results in the absence of sperm at ejaculation. All infertile patients underwent a thorough andrological examination to confirm the diagnosis of azoospermia and to exclude other conditions that could cause infertility, such as cystic fibrosis, orchitis, epididymitis, varicocele, cryptorchidism, obstruction/absence of the vas deferens, chromosomal abnormalities, and Y-chromosome microdeletions. A subgroup of infertile patients who underwent microdissective testicular sperm extraction (mTESE) was also randomly selected from the infertile cohort with the same inclusion criteria and underwent further gene expression analysis.

The research conducted adhered to the highest ethical standards and received approval from the institutional ethics board of Shahid Sadoughi Medical University, identified as IRB No. 1400.233. Furthermore, prior to their involvement, each subject provided informed consent, thereby upholding the principles outlined in the Declaration of Helsinki. All patients provided written informed consent before tissue sampling. The preoperative examination included a medical history, measurement of the testes, semen analysis, and determination of serum levels of follicle-stimulating hormone (FSH), luteinizing hormone (LH), and testosterone. Plasma total cholesterol (T-C) and triglycerides (TG) were measured enzymatically using a Hitachi 912 analyzer (Roche Diagnostics). High-density lipoprotein cholesterol (HDL-C) was measured using a homogeneous method with polyethylene glycol-modified enzymes and alpha-cyclodextrin. Low-density lipoprotein cholesterol (LDL-C) was calculated using the Friedewald equation.

### DNA extraction and amplification-refractory mutation system PCR

Peripheral blood was collected from each participant using EDTA as an anticoagulant and subsequently stored at a temperature of -20°C. Genomic DNA extraction from the blood samples was performed using the QIAamp DNA Blood Midi Kit, following the manufacturer’s provided instructions (Qiagen, Germany). To determine the quantitative concentration of the extracted DNA, a Nanodrop 2000 spectrophotometer was utilized. The amplification refractory mutation system polymerase chain reaction (ARMS-PCR) technique was employed to amplify the specific region of interest within the candidate variants. In this method, oligonucleotides with a mismatched 3’ terminus were avoided, as they are unsuitable as PCR primers. Two separate reactions were carried out using the target DNA, an allele-specific ARMS primer, and a common primer. Agarose gel electrophoresis was used to separate the PCR products, and ethidium bromide staining allowed for visualization of the PCR products under UV irradiation.

### RNA extraction, cDNA synthesis, and realtime-qPCR

Total RNA was extracted from the tissue samples according to the manufacturer’s instructions using the RNeasy plus Universal kit (Qiagen, Hilden, Germany). The concentration and purity of the isolated RNA were assessed using a NanoDrop 2000 spectrophotometer (Thermo Scientific, Wilmington, DE, USA) and confirmed by agarose gel electrophoresis. cDNA synthesis was performed using the Revert Aid First Strand cDNA Synthesis Kit (Thermo Scientific, Vilnius, Lithuania) from 1 µg of total extracted RNA. Each reaction was carried out in an Eppendorf Mastercycler Gradient device (Hamburg, Germany) using a combination of oligo-dT and random hexamer primers. Real-time quantitative polymerase chain reaction (Realtime-qPCR) was performed using an initial denaturation step of 10 min followed by 35 cycles of denaturation at 94 °C for 30 s, annealing at 59 °C for 30 s, and extension at 72 °C for 30 s. To ensure specific amplification of the target gene, a melting curve analysis was conducted by gradually increasing the temperature from 72 °C to 95 °C. Realtime-qPCR reactions were performed in triplicate on 96-well plates (Applied Biosystems), and the average Ct value was calculated for further analysis. Non-template controls (cDNA) were included in all Realtime-qPCR runs to prevent potential contamination. Relative gene expression analysis was carried out using the comparative CT method (2^−ΔΔ^CT) with respect to the housekeeping *ACTB* gene. The features of Realtime-qPCR primers are presented in Table 1.

**Table 1:** Realtime-qPCR and ARMS primers

SNP	Primers	Annealing temperature (°C)	Product size (bp)
rs13306194 Detection of G	R: ACTCAAGTCTTCAATCCTGAAATGTGTC F: TACCTTGTCTTTAGGCTCCATTTTCTG	60	106
rs13306194 Detection of A	R: GACACCTTTTACTTCCCTCTCCTGCA F: GACACCTTTTACTTCCCTCTCCTGCA	60	211
ARMS control primers	R: ACTCAAGTCTTCAATCCTGAAATGTGTC F: TCCTTCCTTCCTTCCTTCCTTTCCTT	60	241
*APOB*	F: GTCAAGACGAGGAAGGGCAA R: GCTCCTTGCAGATGGCTTCT	60	216
*ACTB*	F: CCTTCCTGGGCATGGAG R: CGGAGCAATGATCTTGATC	59	204

### Statistical analysis

We used the Metagenyo on the Web (genepop.curtin.edu.au) web version 4.0 program to calculate the Hardy–Weinberg equilibrium (HWE) for the two groups. We used the chi-squared (χ2) test to determine if there were statistically significant differences in genotype and allele frequencies between the two groups. Calculation of the adjusted odds ratio (OR) and 95% confidence intervals (CI) by comparing the genotype frequency of the study group with that of the control group was used to examine the association between variations and disease risk. Serum levels of LH, testosterone, and FSH, as well as mean testicular volume and age, were examined using an independent t-test. Furthermore, the Kruskal-Wallis test was employed to assess the association of the means of anthropometric measurements and metabolic traits. Statistical analysis was performed using GraphPad Prism 6, *p* ≤ 0.05 was considered significant.

## Results

### Mutual genes

By removing duplicated queries, we obtained 187 obesity-related genes and 195 male infertility-related genes from the OMIM database (Fig. 1a). The *APOB* gene was found in common between the two datasets. List of genes has been included as supplementary material 1.

### Pathogenic nsSNPs

REVEL algorithm identified the rs13306194 variant as potentially pathogenic with a calculated score of 0.524. The rs13306194 (Arg532Trp) was found in the conserved region of protein domain, which can potentially disrupt overall chemical structure and function of the *APOB*.

### Protein-protein network and 3D structure

**Fig. 2:**
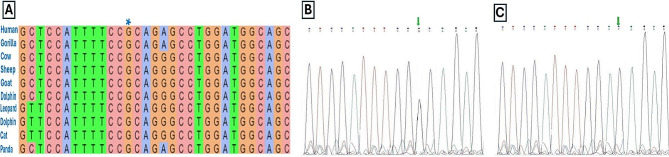
(**A**) Comparative and genetic analysis of allelic variants showcasing an alignment diagram illustrating conservation across species, along with chromatograms of sanger sequencing results for both the (**B**) wild type and (**C**) mutated alleles

STRING analysis revealed a network of proteins closely interacting with APOB, including LPL, *APOA2*, MTTP, *LDLR*, *LPA*, *SORT1*, *APOC3*, *APOA1*, *APOE*, and *CETP* (Fig. 1b). rs13306194 (located in exon 12) was harboring in this homologous model of Lipovitellin with PDB ID ILSH. Both the native and mutant protein models are presented in Fig. 1A. The residue change was R (Arginine) > W (Tryptophan) and their detailed structure of amino acid residue change showed bigger in size, neutrally charged, less hydrophobic properties of mutant residue as compared to wild type positively charged residue (Fig. 2B).

### Clinical parameters

We evaluated the various factors including WHR, hormone levels, and lipid profile, in a sample population of 440 obese individuals (fertile and infertile). The clinical characteristics of the study participants can be found in Table 2. There is a significant association between the WHR, with a p-value of 0.02 and LH with infertility in the obese infertile group, with a p-value of 0.01. In a smaller subset of 42 samples (20 obese fertile and 22 obese infertile) that underwent mTESE for sperm retrieval, no significant differences in average age or testosterone levels were observed between the fertile and infertile obese groups. Interestingly, the infertile obese patients exhibited the highest mean FSH and LH levels. In addition to the observed differences in *APOB* expression, left and right testicular volume were found to have a lower mean value in the infertile obese infertile group compared to the fertile obese group (Table 2).

**Table 2:** Demographic and clinical characteristics of studied individuals

Characteristic	Total Participants		*P* value	mTESE Participants		*P* value
	Obese fertile (*n* = 240)	Obese infertile (*n* = 200)		Obese (*n* = 20)	Non obese (*n* = 22)	
Age (years)	31.2 ± 6.1	40.5 ± 8.3	0.06	39.7 ± 5.4	38.5 ± 9.1	0.07
Body mass index (kg/m2)	27.6 ± 2.3	28.2 ± 1.12	0.82	22.4 ± 3.1	25.9 ± 2.3	0.11
Waist circumference (cm)	67.5 ± 10.0	65.8 ± 6.9	0.19	67.5 ± 10.2	65.8 ± 6.9	0.06
WHR	94.8 ± 7.9	103.8 ± 10.3	0.02*	94.8 ± 7.9	105.1 ± 8.4	0.06
Right testicle volume (ml)	12.5 ± 2.3	13.1 ± 4.1	0.17	10.2 ± 1.3	12.1 ± 3.1	0.02*
Left testicle volume (ml)	12.8 ± 2.2	13.2 ± 2.3	0.27	10.8 ± 1.2	13.0 ± 1.4	0.04*
LH (mIU/mL)	6.3 ± 1.1	7.9 ± 2.9	0.01*	6.4 ± 1.6	8.0 ± 0.6	0.01*
FSH (mIU/mL)	7.4 ± 1.9	8.3 ± 2.1	0.07	6.1 ± 1.2	7.7 ± 1.1	0.03*
Testosterone (ng/mL)	4.3 ± 0.2	5.3 ± 0.8	0.11	4.8 ± 0.3	5.1 ± 0.5	0.11
Total Cholesterol (mg/dl)	222.3 ± 45.3	224.1 ± 38.4	0.06	230.3 ± 35.3	214.1 ± 30.4	0.06
Triglycerides (mg/dl)	178.8 ± 28.8	165.9 ± 46.5	0.27	178.8 ± 30.2	170.2 ± 38.3	0.07
VLDL (mg/dl)	31.6 ± 4.8	34.2 ± 3.8	0.06	31.6 ± 4.8	32.2 ± 6.8	0.11
HDL-C (mg/dl)	42.8 ± 7.6	44.5 ± 14.0	0.11	41.8 ± 5.6	42.9 ± 10.0	0.17
LDL-C (mg/dl)	147.0 ± 39.6	142.7 ± 43.63	0.06	141.0 ± 30.3	138.7 ± 32.9	0.27

Testicular volumes have been measured by Caliper measurement. Asterisk shows significant P values (*P* < 0.05). LH: Luteinizing Hormone, FSH: Follicle-Stimulating Hormone, VLDL: Very Low-Density Lipoprotein, HDL-C High-Density Lipoprotein Cholesterol, LDL-C: Low-Density Lipoprotein Cholesterol, WHR: Waist-to-Hip Ratio

### Genotype and allelic distribution

Table 3 shows the distribution of genotypes for the *APOB* rs13306194 variant in obese infertile and fertile men. The genotype and allele frequencies in non-obese individuals were in Hardy-Weinberg equilibrium. There was a significant difference in the distribution of genotypes and alleles between two groups. Among the infertile group, individuals carrying the AA genotype (P value = 0.001) and A allele (P value = 0.003). These findings suggest that the AA genotype and A allele of the *APOB* gene are associated with infertility in obese men. The SNP was validated by sequencing the PCR products from at least two independent PCR reactions. This was done to rule out PCR artifacts and ensure the accuracy and reliability of the results.

**Table 3:** Genotypes frequencies rs13306194

SNP		Obese fertile (*n* = 240)	Obese infertile (*n* = 200)	OR(95% CI)	*P* Value
Genotype	GG GA AA	106 (44.1) 113 (47.1) 21 (8.8)	73 (36.5) 86 (43.0) 41 (20.5)	(Reference) 1.12 (0.73–1.66) 2.84 (1.55–5.19)	- 0.631 0.001*
Allele	G A	325 (67.7) 155 (32.3)	232 (58.0) 168 (42.0)	(Reference) 1.52 (1.15–2.00)	- 0.003*

SNP: Single nucleotide polymorphism, OR: odd ratio, CI: confidence interval, HWE: Hardy-Weinberg equilibrium

### Expression analysis of APOB

**Fig. 3:**
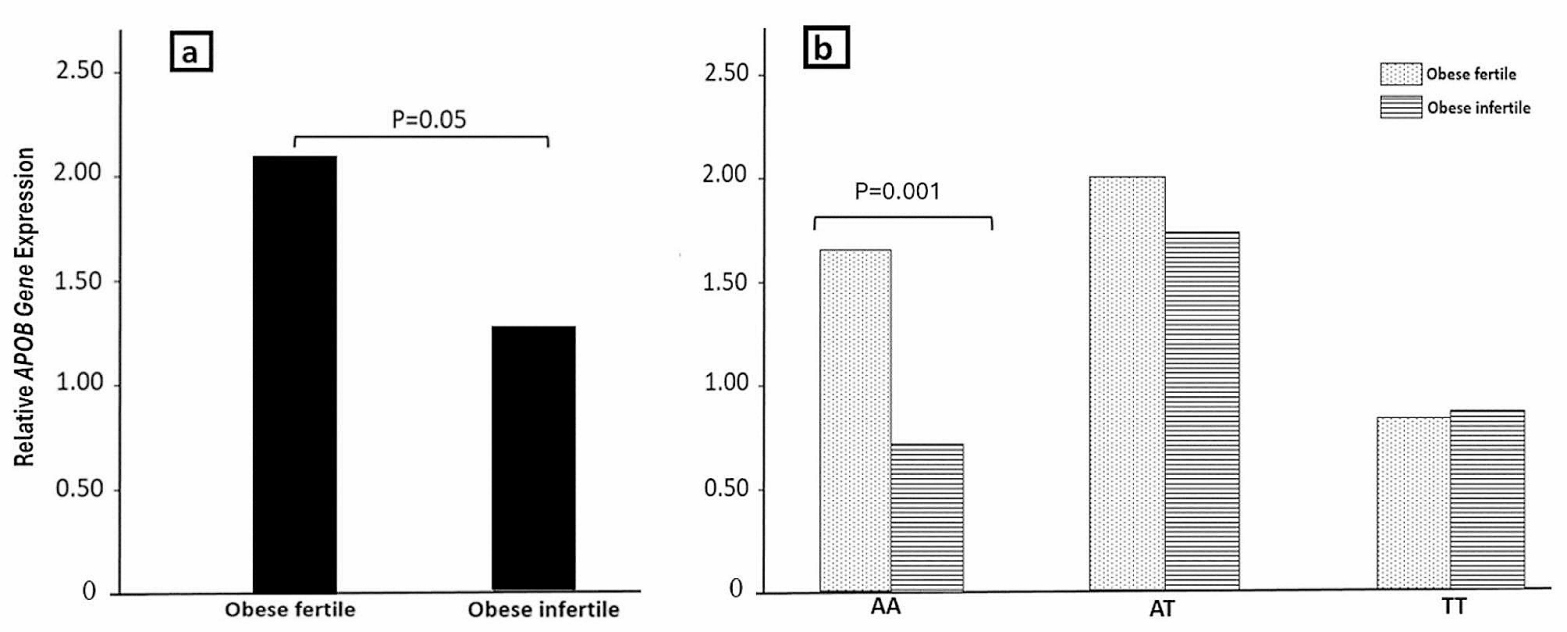
APOB mRNA expression levels showed significantly lower expression in the infertile group compared to the fertile group (**a**). The AA genotype of rs13306194 APOB was associated with a significant decrease in APOB gene expression in obese infertile men (**b**)

The analysis of sperm retrieval success rates revealed that 20 and 16 patients achieved successful sperm retrieval for obese fertile and infertile subjects, respectively (Table 2). *APOB* mRNA expression levels in testicular biopsy samples were determined using qPCR, and the results indicated significantly lower expression in the infertile group compared to the fertile group (*p* < 0.01) (Fig. 3a). Notably, the AA genotype of rs13306194 *APOB* was associated with a significant decrease in APOB gene expression in obese infertile men (Fig. 3b).

## Discussion

We identified 187 obesity-related genes and 195 male infertility-related genes from the OMIM database, with the Apolipoproteins B (*APOB*) gene shared between the two datasets. APOB plays central roles in lipoprotein metabolism and plasma lipid transport as it is essential for the assembly of very-low-density lipoprotein (VLDL) in the liver and intestine. In humans, mutations present in *APOB* lead to familial hypobetalipoproteinemia [22]. The *apoB* gene has been found to be expressed in the testes and epididymis of mice, and research has demonstrated that the expression of human APOB in these tissues can contribute to the correction of infertility issues in mice with a compromised *Apob* gene [23]. Specifically, Huang et al. showed that introducing a human APOB transgene into *apoB*+/− mice resulted in the restoration of normal fertility [24]. This suggests that the APOB gene plays a crucial role in male reproductive health and that its expression in the testes and epididymis is essential for proper fertility.

By scrutinizing all SNPs using the REVEL algorithm, the researchers detected the rs13306194 variant as a probable pathogenic variant with a score of 0.524. The present study observes a notable association between the AA genotype and A allele of the rs13306194 *APOB* gene, and the elevated risk of infertility in men who are obese. This is the first report of the association between rs13306194 in obese infertile patients. The *APOB* rs13306194 variant has been reported to be associated to many metabolic and cardiology diseases. Jang et al. by analyzing whole-genome sequence data of 1478 Taiwanese identified the independent associations between the *APOB* rs13306194 variant and various lipid parameters, including total cholesterol, LDL, non-HDL, triglycerides, and remnant cholesterol [25]. Subsequent studies in Korea also found this variant in individuals with very low LDL cholesterol levels and in a larger Korean population sample [26].

In regards to male infertility, one study by Peterlin et al. found an association between a signal peptide deletion polymorphism in the *APOB* gene and oligoastrostenozoospermia (OAT) in Slovene white men [27]. However, a subsequent study conducted on Indian men did not find any association between the same polymorphism and infertility in any of infertility and sub infertility phenotypes (azoospermia, oligozoospermia, and OAT) [28]. Maurya et al. also identified *Apob* gene as a novel contributor to sperm motility, highlighting its potential role in addressing male infertility. Furthermore, their analysis revealed a strong interaction between the *Apob* gene and glycolytic proteins, suggesting a possible link between energy metabolism and sperm motility [20].

Our findings reveal a novel association between differential *APOB* expression and male infertility. As depicted in Fig. 3a, *APOB* expression levels were markedly lower in obese infertile men compared to fertile controls (*p* < 0.05). Furthermore, the AA genotype of rs13306194 *APOB* was significantly associated with reduced *APOB* gene expression in obese infertile men (Fig. 2b, *p* = 0.05), suggesting a potential role for this genotype in the pathogenesis of male infertility. The observed association between *APOB* expression and the AA genotype of rs13306194 suggests that this genetic alteration may influence *APOB* gene expression and function. Further functional studies are warranted to elucidate the precise mechanism by which this genotype affects gene expression.

Since *APOB* plays a crucial role in lipid metabolism, these findings underscore the critical role of lipid metabolism in male fertility, as lipids are essential components of the sperm cell membrane and liquid content, comprising polyunsaturated fatty acids, plasmalogens, phospholipids, cholesterol, and sphingomyelin. This suggests that maintaining healthy lipid metabolism may be crucial for maintaining male fertility. During sperm activation, capacitation, and acrosome reaction, the lipid composition of the membrane and liquid content undergoes dynamic changes, allowing the sperm to successfully fertilize [29]. Furthermore, reactive oxygen species can damage lipid membranes through peroxidation, leading to sperm DNA fragmentation and impaired motility and morphology, making fertilization more difficult [30].

The HOPE tool was used to analyze the effect of a specific SNP (rs13306194) on the structure of the *APOB* protein. The study found that this SNP is located in a critical and conserved region of the protein that is important for its function. The substitution caused by the SNP may disrupt interactions with other molecules, affect hydrogen bonds, and cause protein misfolding, which could impair LDL-cholesterol metabolism and lipid transport from the liver to other organs. Moreover, it is of great significance to acknowledge that interactions with other genes pertinent to lipid metabolic pathways could potentially impact the male infertility phenotype.

The STRING algorithm analysis identified several genes closely related to APOB that may actively contribute to male infertility, including *APOC3*, *APOA1*, *LDLR*, *LPL*, *APOA2*, *MTTP*, *LPA*, and *SORT1*. *LPL* is essential for steroidogenesis and proper sperm membrane lipid release [31], while *LDLR* and *APOE* knockout have been associated with disturbed testosterone production [32, 33]. The *NTSR3* (*SORT1*) gene is a receptor whose expression positively impacts sperm motility, capacitation in mice, and acrosome reaction in primates [34]. Furthermore, research has highlighted the importance of genes such as *APOA1* and *APOA2* in the defense mechanisms of fish genital tracts, as well as their role in sperm membrane integrity and release of cholesterol [35]. Other lipid metabolism-related genes such as *CETP* and *APOC1* have also been linked to sperm membrane consistency and sperm maturation efficiency in [36]. These recent findings highlight the importance of lipid metabolism-related genes in male infertility and identify potential targets for further research and therapeutic interventions.

Gene-disease and gene-function annotations can be used to explore the relationship between different diseases. This approach can help researchers gain insight into the causes of diseases, identify common pathophysiology, and suggest appropriate treatments that can be applied to multiple diseases [12]. Various methods have been used to search for similarities between diseases, such as examining shared genes, pathways, or functional modules [13]. While these approaches have different levels of accuracy in identifying disease similarities, using a candidate gene approach has been shown to be effective in identifying causative nodes that are connected to multiple diseases. Suthram and colleagues found genetic similarity between diseases plays a significant role in the observed molecular pathological disease similarity. They noted that that diseases which are significantly associated through mRNA expression data and the human protein interaction network also significantly share disease genes [12].

A significant association between WHR and infertility was observed in our study. While there is limited research exploring the relationship between male infertility and WHR, previous studies have presented inconclusive findings regarding the associations between Body Mass Index (BMI) and WHR with subfertility. *One study linked being underweight (BMI < 18.5) with higher rates of azoospermia and lower sperm concentration compared to men of normal weight.* According to Keszthelyi et al.‘s research, the negative relationship between WHR and progressive motility, as well as between WHR and total sperm count, was more robust than the connection between BMI and these parameters [37]. This finding diverges from the report by Wang et al., who found a weak association between BMI and sperm progressive motility, while failing to identify any relationship between WHR and semen parameters. This suggests that the relationship between body composition and male fertility is not fully understood. Fejes et al. [38] pointed out that while WHR itself was not directly associated with changes in fertility markers in a small sample, other research suggests that abdominal fat may indirectly affect fertility through its influence on sex steroids [39], which are predominantly produced in abdominal adipose tissue. Our paper highlights the potential importance of considering WHR in the assessment of male fertility, particularly in the context of obesity.

There was a statistically significant decrease in LH levels in obese infertile group. Numerous studies have emphasized the significance of LH in male reproductive function. Recently, molecular variants of LH have been discovered and associated with male infertility [40]. In line with our results, Amjad et al. showed that LH was associated with male infertility and it was significantly lower in obese patients [41]. Similarly, Farooq et al.‘s research outcome found a positive association in sex hormones with overweight men experiencing infertility [42]. Sertoli cells provide both physical and nutritional support to developing germ cells through direct contact, with developing germ cells adhering to Sertoli cells [43]. Sertoli cell activity is primarily regulated by testosterone and FSH/LH ratios, so decreasing LH levels affect spermatids and decrease sperm counts [44]. Consequently, the reduction in LH level among infertile men could be attributed to the role of hormones in spermatogenesis. Our findings regarding testosterone levels align with previous reports, suggesting that while testosterone may not directly indicate fertility status, the disruption in spermatogenesis and subsequent infertility might still occur due to the altered levels of FSH and LH [45–47]. This could be due to compensatory mechanisms or differing sensitivities of LH and testosterone to the metabolic factors associated with obesity [48].

Male obesity has been linked to poorer IVF outcomes, with studies showing that overweight or obese men have fewer available and high-quality embryos, and reduced fertilization and normal fertilization rates, highlighting the importance of considering obesity factor in IVF procedure [49, 50]. Further research is warranted to fully understand the complex relationships between male obesity, fertility, and the health of subsequent generations, particularly in light of the recent discoveries regarding the role of sperm in transmitting the negative effects of obesity and the promising finding that diet and exercise interventions may help mitigate these effects [51]. To better understand the effects of rs13306194 on male infertility in obese patients, it would also be beneficial to consider parental age in future recruitment, as Opstal and colleagues found an inverse association between paternal age and embryo quality that remained significant even after controlling for maternal age and BMI of both parents [52]. Having knowledge on these facets of male infertility can aid in providing suitable counseling and strategies to modify behavior.

The strength of this study lies in its novel approach of comparing infertility in obese participants with a large sample size. Our study’s novelty lies in examining azoospermic patients with APOB variants, highlighting NOA as a common form of infertility and allowing for a deeper exploration of APOB’s impact on spermatogenesis and related genetic influences. This study contributes to the understanding of the relationship between genetics and obesity-related male infertility, but several limitations should be taken into account. The sample size, though adequate for the purposes of the study, may not be representative of the larger population, potentially limiting the generalizability of the findings. We acknowledge that the inclusion of additional control groups would have enhanced the robustness of our study. However, due to funding constraints, this was not feasible. We emphasize the need for future research to incorporate expanded control groups to strengthen the findings. The exclusion of the female factor represents another limitation of our study, as it restricts our understanding to male-specific infertility, highlighting the need for future research to encompass both partners for a more comprehensive analysis. Furthermore, the study focuses primarily on genetic factors, neglecting the possible influences of environmental and lifestyle factors, such as diet and physical activity [53], on the relationship between genetics and obesity-related male infertility.

In sum, our study identified a potential association between the APOB rs13306194 variant and male infertility in obese patients. Our observations suggest that lipid metabolism plays a crucial role in male reproductive health and that the APOB gene is essential for proper fertility. Our findings also highlight the importance of considering WHR and LH levels in the assessment of male fertility, particularly in the context of obesity. Further research is warranted to fully understand the complex relationships between obesity and male infertility.

### Electronic supplementary material

Below is the link to the electronic supplementary material.

Supplementary Material 1

## Data Availability

Data is provided within the manuscript or supplementary information files.

## Abbreviations

SNPs: Single nucleotide polymorphisms
WHR: Waist-to-Hip Ratio
BMI: Body mass index
OMIM: Online Mendelian Inheritance in Man
MAF: Minor allele frequency
FSH: Follicle-stimulating hormone
LH: Luteinizing hormone
HDL-C: High-density lipoprotein cholesterol
LDL-C: Low-density lipoprotein cholesterol
HWE: Hardy–Weinberg equilibrium
OR: Odds ratio
CI: Confidence intervals
VLDL: Very-low-density lipoprotein
NOA: Non-obstructive azoospermia
