# Supplementary material for: Where do obesity and male infertility collide?
Source: BMC Med Genomics. 2024 May 10;17:128. doi: 10.1186/s12920-024-01897-5 (PMC11088066; doi:10.1186/s12920-024-01897-5)
Supplement: Supplementary file 1 — Supplementary Material 1 [file 12920_2024_1897_MOESM1_ESM.docx]

**Obesity genes retrieved OMIM database**

| Approved symbol | Approved name | Chromosome | Chromosome location | NCBI gene ID | OMIM ID |
| --- | --- | --- | --- | --- | --- |
| ACOT11 | acyl-CoA thioesterase 11 | 1 | 1p32.3 | 26027 | 606803 |
| ACVR1C | activin A receptor type 1C | 2 | 2q24.1 | 130399 | 608981 |
| ADCY3 | adenylate cyclase 3 | 2 | 2p23.3 | 109 | 600291 |
| ADIPOQ | adiponectin, C1Q and collagen domain containing | 3 | 3q27.3 | 9370 | 605441 |
| ADIPOR1 | adiponectin receptor 1 | 1 | 1q32.1 | 51094 | 607945 |
| ADRA2B | adrenoceptor alpha 2B | 2 | 2q11.2 | 151 | 104260 |
| ADRB2 | adrenoceptor beta 2 | 5 | 5q32 | 154 | 109690 |
| AFF4 | ALF transcription elongation factor 4 | 5 | 5q31.1 | 27125 | 604417 |
| AGL | amylo-alpha-1, 6-glucosidase, 4-alpha-glucanotransferase | 1 | 1p21.2 | 178 | 610860 |
| AGT | angiotensinogen | 1 | 1q42.2 | 183 | 106150 |
| ALB | albumin | 4 | 4q13.3 | 213 | 103600 |
| ALMS1 | ALMS1 centrosome and basal body associated protein | 2 | 2p13.1 | 7840 | 606844 |
| AMY1A | amylase alpha 1A | 1 | 1p21.1 | 276 | 104700 |
| AMY1B | amylase alpha 1B | 1 | 1p21.1 | 277 | 104701 |
| AMY1C | amylase alpha 1C | 1 | 1p21.1 | 278 | 104702 |
| AMY2A | amylase alpha 2A | 1 | 1p21.1 | 279 | 104650 |
| AMY2B | amylase alpha 2B | 1 | 1p21.1 | 280 | 104660 |
| ANGPTL7 | angiopoietin like 7 | 1 | 1p36.22 | 10218 | 618517 |
| APOB | apolipoprotein B | 2 | 2p24.1 | 338 | 107730 |
| ARL13B | ADP ribosylation factor like GTPase 13B | 3 | 3q11.1-q11.2 | 200894 | 608922 |
| ATP10D | ATPase phospholipid transporting 10D (putative) | 4 | 4p12 | 57205 | 619815 |
| BBS5 | Bardet-Biedl syndrome 5 | 2 | 2q31.1 | 129880 | 603650 |
| BBS7 | Bardet-Biedl syndrome 7 | 4 | 4q27 | 55212 | 607590 |
| BBS12 | Bardet-Biedl syndrome 12 | 4 | 4q27 | 166379 | 610683 |
| BECN2 | beclin 2 | 1 | 1q43 | 441925 | 615687 |
| BGLAP | bone gamma-carboxyglutamate protein | 1 | 1q22 | 632 | 112260 |
| C1QTNF2 | C1q and TNF related 2 | 5 | 5q33.3 | 114898 | 618647 |
| C1QTNF3 | C1q and TNF related 3 | 5 | 5p13.2 | 114899 | 612045 |
| CAPN10 | calpain 10 | 2 | 2q37.3 | 11132 | 605286 |
| CARTPT | CART prepropeptide | 5 | 5q13.2 | 9607 | 602606 |
| CCDC80 | coiled-coil domain containing 80 | 3 | 3q13.2 | 151887 | 608298 |
| CCK | cholecystokinin | 3 | 3p22.1 | 885 | 118440 |
| CCKAR | cholecystokinin A receptor | 4 | 4p15.2 | 886 | 118444 |
| CELA2A | chymotrypsin like elastase 2A | 1 | 1p36.21 | 63036 | 609443 |
| CEP19 | centrosomal protein 19 | 3 | 3q29 | 84984 | 615586 |
| CFH | complement factor H | 1 | 1q31.3 | 3075 | 134370 |
| CIDEC | cell death inducing DFFA like effector c | 3 | 3p25.3 | 63924 | 612120 |
| CLOCK | clock circadian regulator | 4 | 4q12 | 9575 | 601851 |
| COP1 | COP1 E3 ubiquitin ligase | 1 | 1q25.1-q25.2 | 64326 | 608067 |
| CPE | carboxypeptidase E | 4 | 4q32.3 | 1363 | 114855 |
| CRP | C-reactive protein | 1 | 1q23.2 | 1401 | 123260 |
| CRTC2 | CREB regulated transcription coactivator 2 | 1 | 1q21.3 | 200186 | 608972 |
| CXCL8 | C-X-C motif chemokine ligand 8 | 4 | 4q13.3 | 3576 | 146930 |
| DCP2 | decapping mRNA 2 | 5 | 5q22.2 | 167227 | 609844 |
| DEGS1 | delta 4-desaturase, sphingolipid 1 | 1 | 1q42.11 | 8560 | 615843 |
| DGKD | diacylglycerol kinase delta | 2 | 2q37.1 | 8527 | 601826 |
| DNMT3A | DNA methyltransferase 3 alpha | 2 | 2p23.3 | 1788 | 602769 |
| DPP4 | dipeptidyl peptidase 4 | 2 | 2q24.2 | 1803 | 102720 |
| EIF2AK3 | eukaryotic translation initiation factor 2 alpha kinase 3 | 2 | 2p11.2 | 9451 | 604032 |
| ELOVL6 | ELOVL fatty acid elongase 6 | 4 | 4q25 | 79071 | 611546 |
| ERFE | erythroferrone | 2 | 2q37.3 | 151176 | 615099 |
| EXOC6B | exocyst complex component 6B | 2 | 2p13.2 | 23233 | 607880 |
| F2RL1 | F2R like trypsin receptor 1 | 5 | 5q13.3 | 2150 | 600933 |
| F3 | coagulation factor III, tissue factor | 1 | 1p21.3 | 2152 | 134390 |
| F5 | coagulation factor V | 1 | 1q24.2 | 2153 | 612309 |
| F12 | coagulation factor XII | 5 | 5q35.3 | 2161 | 610619 |
| FAAH | fatty acid amide hydrolase | 1 | 1p33 | 2166 | 602935 |
| FABP2 | fatty acid binding protein 2 | 4 | 4q26 | 2169 | 134640 |
| FAIM | Fas apoptotic inhibitory molecule | 3 | 3q22.3 | 55179 | 617535 |
| FBXO11 | F-box protein 11 | 2 | 2p16.3 | 80204 | 607871 |
| FGA | fibrinogen alpha chain | 4 | 4q31.3 | 2243 | 134820 |
| FNDC5 | fibronectin type III domain containing 5 | 1 | 1p35.1 | 252995 | 611906 |
| FOXP1 | forkhead box P1 | 3 | 3p13 | 27086 | 605515 |
| FXR1 | FMR1 autosomal homolog 1 | 3 | 3q26.33 | 8087 | 600819 |
| GATA2 | GATA binding protein 2 | 3 | 3q21.3 | 2624 | 137295 |
| GCG | glucagon | 2 | 2q24.2 | 2641 | 138030 |
| GCKR | glucokinase regulator | 2 | 2p23.3 | 2646 | 600842 |
| GGPS1 | geranylgeranyl diphosphate synthase 1 | 1 | 1q42.3 | 9453 | 606982 |
| GHRL | ghrelin and obestatin prepropeptide | 3 | 3p25.3 | 51738 | 605353 |
| GHSR | growth hormone secretagogue receptor | 3 | 3q26.31 | 2693 | 601898 |
| GPAT3 | glycerol-3-phosphate acyltransferase 3 | 4 | 4q21.23 | 84803 | 610958 |
| GPBAR1 | G protein-coupled bile acid receptor 1 | 2 | 2q35 | 151306 | 610147 |
| GPD2 | glycerol-3-phosphate dehydrogenase 2 | 2 | 2q24.1 | 2820 | 138430 |
| GPR45 | G protein-coupled receptor 45 | 2 | 2q12.1 | 11250 | 604838 |
| H6PD | hexose-6-phosphate dehydrogenase/glucose 1-dehydrogenase | 1 | 1p36.22 | 9563 | 138090 |
| HDAC3 | histone deacetylase 3 | 5 | 5q31.3 | 8841 | 605166 |
| HLA-G | major histocompatibility complex, class I, G | 6 | 6p22.1 | 3135 | 142871 |
| HNMT | histamine N-methyltransferase | 2 | 2q22.1 | 3176 | 605238 |
| HRG | histidine rich glycoprotein | 3 | 3q27.3 | 3273 | 142640 |
| HSD11B1 | hydroxysteroid 11-beta dehydrogenase 1 | 1 | 1q32.2 | 3290 | 600713 |
| IFT172 | intraflagellar transport 172 | 2 | 2p23.3 | 26160 | 607386 |
| IGF2BP2 | insulin like growth factor 2 mRNA binding protein 2 | 3 | 3q27.2 | 10644 | 608289 |
| IKBKE | inhibitor of nuclear factor kappa B kinase subunit epsilon | 1 | 1q32.1 | 9641 | 605048 |
| IL1RN | interleukin 1 receptor antagonist | 2 | 2q14.1 | 3557 | 147679 |
| IL6R | interleukin 6 receptor | 1 | 1q21.3 | 3570 | 147880 |
| IL13 | interleukin 13 | 5 | 5q31.1 | 3596 | 147683 |
| IL17RC | interleukin 17 receptor C | 3 | 3p25.3 | 84818 | 610925 |
| INSIG2 | insulin induced gene 2 | 2 | 2q14.1-q14.2 | 51141 | 608660 |
| IRS1 | insulin receptor substrate 1 | 2 | 2q36.3 | 3667 | 147545 |
| KCNA3 | potassium voltage-gated channel subfamily A member 3 | 1 | 1p13.3 | 3738 | 176263 |
| KDM3A | lysine demethylase 3A | 2 | 2p11.2 | 55818 | 611512 |
| KDR | kinase insert domain receptor | 4 | 4q12 | 3791 | 191306 |
| KIDINS220 | kinase D interacting substrate 220 | 2 | 2p25.1 | 57498 | 615759 |
| KNG1 | kininogen 1 | 3 | 3q27.3 | 3827 | 612358 |
| LEAP2 | liver enriched antimicrobial peptide 2 | 5 | 5q31.1 | 116842 | 611373 |
| LEPR | leptin receptor | 1 | 1p31.3 | 3953 | 601007 |
| LEPROT | leptin receptor overlapping transcript | 1 | 1p31.3 | 54741 | 613461 |
| LMNA | lamin A/C | 1 | 1q22 | 4000 | 150330 |
| LPIN1 | lipin 1 | 2 | 2p25.1 | 23175 | 605518 |
| LYST | lysosomal trafficking regulator | 1 | 1q42.3 | 1130 | 606897 |
| LZTFL1 | leucine zipper transcription factor like 1 | 3 | 3p21.31 | 54585 | 606568 |
| MAPK9 | mitogen-activated protein kinase 9 | 5 | 5q35.3 | 5601 | 602896 |
| MFN2 | mitofusin 2 | 1 | 1p36.22 | 9927 | 608507 |
| MIA3 | MIA SH3 domain ER export factor 3 | 1 | 1q41 | 375056 | 613455 |
| MIR103A1 | microRNA 103a-1 | 5 | 5q34 | 406895 | 613187 |
| MOG | myelin oligodendrocyte glycoprotein | 6 | 6p22.1 | 4340 | 159465 |
| MSTN | myostatin | 2 | 2q32.2 | 2660 | 601788 |
| MTTP | microsomal triglyceride transfer protein | 4 | 4q23 | 4547 | 157147 |
| MUC7 | mucin 7, secreted | 4 | 4q13.3 | 4589 | 158375 |
| MYT1L | myelin transcription factor 1 like | 2 | 2p25.3 | 23040 | 613084 |
| NCOA1 | nuclear receptor coactivator 1 | 2 | 2p23.3 | 8648 | 602691 |
| NEUROD1 | neuronal differentiation 1 | 2 | 2q31.3 | 4760 | 601724 |
| NFKB1 | nuclear factor kappa B subunit 1 | 4 | 4q24 | 4790 | 164011 |
| NHLH2 | nescient helix-loop-helix 2 | 1 | 1p13.1 | 4808 | 162361 |
| NKX1-1 | NK1 homeobox 1 | 4 | 4p16.3 | 54729 | 617869 |
| NKX2-5 | NK2 homeobox 5 | 5 | 5q35.1 | 1482 | 600584 |
| NLRP3 | NLR family pyrin domain containing 3 | 1 | 1q44 | 114548 | 606416 |
| NMU | neuromedin U | 4 | 4q12 | 10874 | 605103 |
| NOCT | nocturnin | 4 | 4q31.1 | 25819 | 608468 |
| NPFFR2 | neuropeptide FF receptor 2 | 4 | 4q13.3 | 10886 | 607449 |
| NPY5R | neuropeptide Y receptor Y5 | 4 | 4q32.2 | 4889 | 602001 |
| NPY6R | neuropeptide Y receptor Y6 (pseudogene) | 5 | 5q31.2 | 4888 | 601770 |
| NR1D2 | nuclear receptor subfamily 1 group D member 2 | 3 | 3p24.2 | 9975 | 602304 |
| NR3C1 | nuclear receptor subfamily 3 group C member 1 | 5 | 5q31.3 | 2908 | 138040 |
| NR0B2 | nuclear receptor subfamily 0 group B member 2 | 1 | 1p36.11 | 8431 | 604630 |
| NSD1 | nuclear receptor binding SET domain protein 1 | 5 | 5q35.3 | 64324 | 606681 |
| OGG1 | 8-oxoguanine DNA glycosylase | 3 | 3p25.3 | 4968 | 601982 |
| OMA1 | OMA1 zinc metallopeptidase | 1 | 1p32.2-p32.1 | 115209 | 617081 |
| P4HTM | prolyl 4-hydroxylase, transmembrane | 3 | 3p21.31 | 54681 | 614584 |
| PBRM1 | polybromo 1 | 3 | 3p21.1 | 55193 | 606083 |
| PCSK1 | proprotein convertase subtilisin/kexin type 1 | 5 | 5q15 | 5122 | 162150 |
| PDE4D | phosphodiesterase 4D | 5 | 5q11.2-q12.1 | 5144 | 600129 |
| PDE11A | phosphodiesterase 11A | 2 | 2q31.2 | 50940 | 604961 |
| PGRMC2 | progesterone receptor membrane component 2 | 4 | 4q28.2 | 10424 | 607735 |
| PKDCC | protein kinase domain containing, cytoplasmic | 2 | 2p21 | 91461 | 614150 |
| PLA2G2E | phospholipase A2 group IIE | 1 | 1p36.13 | 30814 | 618320 |
| PM20D1 | peptidase M20 domain containing 1 | 1 | 1q32.1 | 148811 | 617124 |
| PNRC2 | proline rich nuclear receptor coactivator 2 | 1 | 1p36.11 | 55629 | 611882 |
| POMC | proopiomelanocortin | 2 | 2p23.3 | 5443 | 176830 |
| PPARG | peroxisome proliferator activated receptor gamma | 3 | 3p25.2 | 5468 | 601487 |
| PPARGC1A | PPARG coactivator 1 alpha | 4 | 4p15.2 | 10891 | 604517 |
| PPARGC1B | PPARG coactivator 1 beta | 5 | 5q32 | 133522 | 608886 |
| PRDM16 | PR/SET domain 16 | 1 | 1p36.32 | 63976 | 605557 |
| PROK2 | prokineticin 2 | 3 | 3p13 | 60675 | 607002 |
| PROP1 | PROP paired-like homeobox 1 | 5 | 5q35.3 | 5626 | 601538 |
| PROX1 | prospero homeobox 1 | 1 | 1q32.3 | 5629 | 601546 |
| PTGER3 | prostaglandin E receptor 3 | 1 | 1p31.1 | 5733 | 176806 |
| PTGS2 | prostaglandin-endoperoxide synthase 2 | 1 | 1q31.1 | 5743 | 600262 |
| PTPRF | protein tyrosine phosphatase receptor type F | 1 | 1p34.2 | 5792 | 179590 |
| RBP1 | retinol binding protein 1 | 3 | 3q23 | 5947 | 180260 |
| RETSAT | retinol saturase | 2 | 2p11.2 | 54884 | 617597 |
| RNPC3 | RNA binding region (RNP1, RRM) containing 3 | 1 | 1p21.1 | 55599 | 618016 |
| ROBO1 | roundabout guidance receptor 1 | 3 | 3p12.3 | 6091 | 602430 |
| SCD5 | stearoyl-CoA desaturase 5 | 4 | 4q21.22 | 79966 | 608370 |
| SCGB3A2 | secretoglobin family 3A member 2 | 5 | 5q32 | 117156 | 606531 |
| SDC1 | syndecan 1 | 2 | 2p24.1 | 6382 | 186355 |
| SDC3 | syndecan 3 | 1 | 1p35.2 | 9672 | 186357 |
| SDCCAG8 | SHH signaling and ciliogenesis regulator SDCCAG8 | 1 | 1q43-q44 | 10806 | 613524 |
| SDHA | succinate dehydrogenase complex flavoprotein subunit A | 5 | 5p15.33 | 6389 | 600857 |
| SERTAD2 | SERTA domain containing 2 | 2 | 2p14 | 9792 | 617851 |
| SETD2 | SET domain containing 2, histone lysine methyltransferase | 3 | 3p21.31 | 29072 | 612778 |
| SGIP1 | SH3GL interacting endocytic adaptor 1 | 1 | 1p31.3 | 84251 | 611540 |
| SLC2A2 | solute carrier family 2 member 2 | 3 | 3q26.2 | 6514 | 138160 |
| SLC10A7 | solute carrier family 10 member 7 | 4 | 4q31.22 | 84068 | 611459 |
| SLC25A4 | solute carrier family 25 member 4 | 4 | 4q35.1 | 291 | 103220 |
| SLC25A44 | solute carrier family 25 member 44 | 1 | 1q22 | 9673 | 610824 |
| SLC26A2 | solute carrier family 26 member 2 | 5 | 5q32 | 1836 | 606718 |
| SPEN | spen family transcriptional repressor | 1 | 1p36.21-p36.13 | 23013 | 613484 |
| SPP1 | secreted phosphoprotein 1 | 4 | 4q22.1 | 6696 | 166490 |
| SQSTM1 | sequestosome 1 | 5 | 5q35.3 | 8878 | 601530 |
| SUCLG1 | succinate-CoA ligase GDP/ADP-forming subunit alpha | 2 | 2p11.2 | 8802 | 611224 |
| TBC1D1 | TBC1 domain family member 1 | 4 | 4p14 | 23216 | 609850 |
| THRB | thyroid hormone receptor beta | 3 | 3p24.2 | 7068 | 190160 |
| TLR5 | toll like receptor 5 | 1 | 1q41 | 7100 | 603031 |
| TMEM18 | transmembrane protein 18 | 2 | 2p25.3 | 129787 | 613220 |
| TNF | tumor necrosis factor | 6 | 6p21.33 | 7124 | 191160 |
| TRAF3IP1 | TRAF3 interacting protein 1 | 2 | 2q37.3 | 26146 | 607380 |
| TRIP12 | thyroid hormone receptor interactor 12 | 2 | 2q36.3 | 9320 | 604506 |
| UCN | urocortin | 2 | 2p23.3 | 7349 | 600945 |
| UCP1 | uncoupling protein 1 | 4 | 4q31.1 | 7350 | 113730 |
| USF1 | upstream transcription factor 1 | 1 | 1q23.3 | 7391 | 191523 |
| VHL | von Hippel-Lindau tumor suppressor | 3 | 3p25.3 | 7428 | 608537 |
| WFS1 | wolframin ER transmembrane glycoprotein | 4 | 4p16.1 | 7466 | 606201 |
| XRCC4 | X-ray repair cross complementing 4 | 5 | 5q14.2 | 7518 | 194363 |
| ZBTB20 | zinc finger and BTB domain containing 20 | 3 | 3q13.31 | 26137 | 606025 |
| ZFP57 | ZFP57 zinc finger protein | 6 | 6p22.1 | 346171 | 612192 |
| ZFP69 | ZFP69 zinc finger protein | 1 | 1p34.2 | 339559 | 617939 |

**Male infertility genes retrieved OMIM database**

| Approved symbol | Approved name | Chromosome | Chromosome location | OMIM ID | NCBI gene ID |
| --- | --- | --- | --- | --- | --- |
| ABHD16B | abhydrolase domain containing 16B | 20 | 20q13.33 | 620190 | 140701 |
| ACE | angiotensin I converting enzyme | 17 | 17q23.3 | 106180 | 1636 |
| ACR | acrosin | 22 | 22q13.33 | 102480 | 49 |
| ACTL7A | actin like 7A | 9 | 9q31.3 | 604303 | 10881 |
| ACTL9 | actin like 9 | 19 | 19p13.2 | 619251 | 284382 |
| ADAD1 | adenosine deaminase domain containing 1 | 4 | 4q27 | 614130 | 132612 |
| ADGRG2 | adhesion G protein-coupled receptor G2 | X | Xp22.13 | 300572 | 10149 |
| AK7 | adenylate kinase 7 | 14 | 14q32.2 | 615364 | 122481 |
| AK9 | adenylate kinase 9 | 6 | 6q21 | 615358 | 221264 |
| AKAP3 | A-kinase anchoring protein 3 | 12 | 12p13.32 | 604689 | 10566 |
| ANKRD31 | ankyrin repeat domain 31 | 5 | 5q13.3 | 618423 | 256006 |
| APAF1 | apoptotic peptidase activating factor 1 | 12 | 12q23.1 | 602233 | 317 |
| APOB | apolipoprotein B | 2 | 2p24.1 | 107730 | 338 |
| AR | androgen receptor | X | Xq12 | 313700 | 367 |
| ARMC12 | armadillo repeat containing 12 | 6 | 6p21.31 | 620377 | 221481 |
| AURKC | aurora kinase C | 19 | 19q13.43 | 603495 | 6795 |
| BBS1 | Bardet-Biedl syndrome 1 | 11 | 11q13.2 | 209901 | 582 |
| BBS2 | Bardet-Biedl syndrome 2 | 16 | 16q13 | 606151 | 583 |
| BCL2L2 | BCL2 like 2 | 14 | 14q11.2 | 601931 | 599 |
| BCL10 | BCL10 immune signaling adaptor | 1 | 1p22.3 | 603517 | 8915 |
| BMPR1B | bone morphogenetic protein receptor type 1B | 4 | 4q22.3 | 603248 | 658 |
| BPY2 | basic charge Y-linked 2 | Y | Yq11.223 | 400013 | 9083 |
| BRDT | bromodomain testis associated | 1 | 1p22.1 | 602144 | 676 |
| BRME1 | break repair meiotic recombinase recruitment factor 1 | 19 | 19p13.12 | 619276 | 79173 |
| BRWD1 | bromodomain and WD repeat domain containing 1 | 21 | 21q22.2 | 617824 | 54014 |
| C2CD6 | C2 calcium dependent domain containing 6 | 2 | 2q33.1 | 619776 | 151254 |
| CADM1 | cell adhesion molecule 1 | 11 | 11q23.3 | 605686 | 23705 |
| CADM4 | cell adhesion molecule 4 | 19 | 19q13.31 | 609744 | 199731 |
| CAMK4 | calcium/calmodulin dependent protein kinase IV | 5 | 5q22.1 | 114080 | 814 |
| CATIP | ciliogenesis associated TTC17 interacting protein | 2 | 2q35 | 619387 | 375307 |
| CATSPER1 | cation channel sperm associated 1 | 11 | 11q13.1 | 606389 | 117144 |
| CATSPER2 | cation channel sperm associated 2 | 15 | 15q15.3 | 607249 | 117155 |
| CATSPERE | catsper channel auxiliary subunit epsilon | 1 | 1q44 | 617510 | 257044 |
| CCDC34 | coiled-coil domain containing 34 | 11 | 11p14.1 | 612324 | 91057 |
| CCDC39 | coiled-coil domain 39 molecular ruler complex subunit | 3 | 3q26.33 | 613798 | 339829 |
| CCDC62 | coiled-coil domain containing 62 | 12 | 12q24.31 | 613481 | 84660 |
| CCNO | cyclin O | 5 | 5q11.2 | 607752 | 10309 |
| CDAN1 | codanin 1 | 15 | 15q15.2 | 607465 | 146059 |
| CEP112 | centrosomal protein 112 | 17 | 17q24.1 | 618980 | 201134 |
| CETN1 | centrin 1 | 18 | 18p11.32 | 603187 | 1068 |
| CFAP43 | cilia and flagella associated protein 43 | 10 | 10q25.1 | 617558 | 80217 |
| CFAP44 | cilia and flagella associated protein 44 | 3 | 3q13.2 | 617559 | 55779 |
| CFAP45 | cilia and flagella associated protein 45 | 1 | 1q23.2 | 605152 | 25790 |
| CFAP47 | cilia and flagella associated protein 47 | X | Xp21.1 | 301057 | 286464 |
| CFAP52 | cilia and flagella associated protein 52 | 17 | 17p13.1 | 609804 | 146845 |
| CFAP58 | cilia and flagella associated protein 58 | 10 | 10q25.1 | 619129 | 159686 |
| CFAP61 | cilia and flagella associated protein 61 | 20 | 20p11.23 | 620381 | 26074 |
| CFAP65 | cilia and flagella associated protein 65 | 2 | 2q35 | 614270 | 255101 |
| CFAP69 | cilia and flagella associated protein 69 | 7 | 7q21.13 | 617949 | 79846 |
| CFAP70 | cilia and flagella associated protein 70 | 10 | 10q22.2 | 618661 | 118491 |
| CFAP74 | cilia and flagella associated protein 74 | 1 | 1p36.33 | 620187 | 85452 |
| CFAP91 | cilia and flagella associated protein 91 | 3 | 3q13.33 | 609910 | 89876 |
| CFAP251 | cilia and flagella associated protein 251 | 12 | 12q24.31 | 618146 | 144406 |
| CFAP300 | cilia and flagella associated protein 300 | 11 | 11q22.1 | 618058 | 85016 |
| CFTR | CF transmembrane conductance regulator | 7 | 7q31.2 | 602421 | 1080 |
| CKMT1A | creatine kinase, mitochondrial 1A | 15 | 15q15.3 | 613415 | 548596 |
| CKMT1B | creatine kinase, mitochondrial 1B | 15 | 15q15.3 | 123290 | 1159 |
| CLCN2 | chloride voltage-gated channel 2 | 3 | 3q27.1 | 600570 | 1181 |
| CLDN2 | claudin 2 | X | Xq22.3 | 300520 | 9075 |
| CLDN11 | claudin 11 | 3 | 3q26.2 | 601326 | 5010 |
| CLDN16 | claudin 16 | 3 | 3q28 | 603959 | 10686 |
| CSTF2T | cleavage stimulation factor subunit 2 tau variant | 10 | 10q21.1 | 611968 | 23283 |
| CT55 | cancer/testis antigen 55 | X | Xq26.3 | 301105 | 54967 |
| CTNS | cystinosin, lysosomal cystine transporter | 17 | 17p13.2 | 606272 | 1497 |
| DAZ1 | deleted in azoospermia 1 | Y | Yq11.223 | 400003 | 1617 |
| DAZ4 | deleted in azoospermia 4 | Y | Yq11.23 | 400048 | 57135 |
| DAZL | deleted in azoospermia like | 3 | 3p24.3 | 601486 | 1618 |
| DCXR | dicarbonyl and L-xylulose reductase | 17 | 17q25.3 | 608347 | 51181 |
| DDX3Y | DEAD-box helicase 3 Y-linked | Y | Yq11.221 | 400010 | 8653 |
| DMRT1 | doublesex and mab-3 related transcription factor 1 | 9 | 9p24.3 | 602424 | 1761 |
| DNAH1 | dynein axonemal heavy chain 1 | 3 | 3p21.1 | 603332 | 25981 |
| DNAH2 | dynein axonemal heavy chain 2 | 17 | 17p13.1 | 603333 | 146754 |
| DNAH5 | dynein axonemal heavy chain 5 | 5 | 5p15.2 | 603335 | 1767 |
| DNAH7 | dynein axonemal heavy chain 7 | 2 | 2q32.3 | 610061 | 56171 |
| DNAH8 | dynein axonemal heavy chain 8 | 6 | 6p21.2 | 603337 | 1769 |
| DNAH9 | dynein axonemal heavy chain 9 | 17 | 17p12 | 603330 | 1770 |
| DNAH10 | dynein axonemal heavy chain 10 | 12 | 12q24.31 | 605884 | 196385 |
| DNAH11 | dynein axonemal heavy chain 11 | 7 | 7p15.3 | 603339 | 8701 |
| DNAH17 | dynein axonemal heavy chain 17 | 17 | 17q25.3 | 610063 | 8632 |
| DNAJB13 | DnaJ heat shock protein family (Hsp40) member B13 | 11 | 11q13.4 | 610263 | 374407 |
| DNALI1 | dynein axonemal light intermediate chain 1 | 1 | 1p34.3 | 602135 | 7802 |
| DNHD1 | dynein heavy chain domain 1 | 11 | 11p15.4 | 617277 | 144132 |
| DPCD | deleted in primary ciliary dyskinesia homolog (mouse) | 10 | 10q24.32 | 616467 | 25911 |
| DPY19L2 | dpy-19 like 2 | 12 | 12q14.2 | 613893 | 283417 |
| DRC1 | dynein regulatory complex subunit 1 | 2 | 2p23.3 | 615288 | 92749 |
| DZIP1 | DAZ interacting zinc finger protein 1 | 13 | 13q32.1 | 608671 | 22873 |
| EGF | epidermal growth factor | 4 | 4q25 | 131530 | 1950 |
| EMC10 | ER membrane protein complex subunit 10 | 19 | 19q13.33 | 614545 | 284361 |
| ENO4 | enolase 4 | 10 | 10q25.3 | 131375 | 387712 |
| EPB41L2 | erythrocyte membrane protein band 4.1 like 2 | 6 | 6q23.1-q23.2 | 603237 | 2037 |
| FANCM | FA complementation group M | 14 | 14q21.2 | 609644 | 57697 |
| FBXO43 | F-box protein 43 | 8 | 8q22.2 | 609110 | 286151 |
| FCGR2A | Fc gamma receptor IIa | 1 | 1q23.3 | 146790 | 2212 |
| FKBP4 | FKBP prolyl isomerase 4 | 12 | 12p13.33 | 600611 | 2288 |
| FKBP6 | FKBP prolyl isomerase family member 6 (inactive) | 7 | 7q11.23 | 604839 | 8468 |
| FOXI1 | forkhead box I1 | 5 | 5q35.1 | 601093 | 2299 |
| FOXJ2 | forkhead box J2 | 12 | 12p13.31 | 619162 | 55810 |
| FSHR | follicle stimulating hormone receptor | 2 | 2p16.3 | 136435 | 2492 |
| GCNA | germ cell nuclear acidic peptidase | X | Xq13.1 | 300369 | 93953 |
| GGN | gametogenetin | 19 | 19q13.2 | 609966 | 199720 |
| GK2 | glycerol kinase 2 | 4 | 4q21.21 | 600148 | 2712 |
| GMCL1 | germ cell-less 1, spermatogenesis associated | 2 | 2p13.3 | 618627 | 64395 |
| GMNC | geminin coiled-coil domain containing | 3 | 3q28 | 614448 | 647309 |
| GNPAT | glyceronephosphate O-acyltransferase | 1 | 1q42.2 | 602744 | 8443 |
| HAUS7 | HAUS augmin like complex subunit 7 | X | Xq28 | 300540 | 55559 |
| HSPA2 | heat shock protein family A (Hsp70) member 2 | 14 | 14q23.3 | 140560 | 3306 |
| HSPA4L | heat shock protein family A (Hsp70) member 4 like | 4 | 4q28.1 | 619077 | 22824 |
| IFT74 | intraflagellar transport 74 | 9 | 9p21.2 | 608040 | 80173 |
| IQCN | IQ motif containing N | 19 | 19p13.11 | 620160 | 80726 |
| IQUB | IQ motif and ubiquitin domain containing | 7 | 7q31.32 | 620557 | 154865 |
| KASH5 | KASH domain containing 5 | 19 | 19q13.33 | 618125 | 147872 |
| KATNB1 | katanin regulatory subunit B1 | 16 | 16q21 | 602703 | 10300 |
| KCNU1 | potassium calcium-activated channel subfamily U member 1 | 8 | 8p11.23 | 615215 | 157855 |
| KHDRBS1 | KH RNA binding domain containing, signal transduction associated 1 | 1 | 1p35.2 | 602489 | 10657 |
| KIT | KIT proto-oncogene, receptor tyrosine kinase | 4 | 4q12 | 164920 | 3815 |
| KLHL10 | kelch like family member 10 | 17 | 17q21.2 | 608778 | 317719 |
| LHB | luteinizing hormone subunit beta | 19 | 19q13.33 | 152780 | 3972 |
| M1AP | meiosis 1 associated protein | 2 | 2p13.1 | 619098 | 130951 |
| MAGEB4 | MAGE family member B4 | X | Xp21.2 | 300153 | 4115 |
| MCIDAS | multiciliate differentiation and DNA synthesis associated cell cycle protein | 5 | 5q11.2 | 614086 | 345643 |
| MDC1 | mediator of DNA damage checkpoint 1 | 6 | 6p21.33 | 607593 | 9656 |
| MEIOB | meiosis specific with OB-fold | 16 | 16p13.3 | 617670 | 254528 |
| MNS1 | meiosis specific nuclear structural 1 | 15 | 15q21.3 | 610766 | 55329 |
| MOV10L1 | Mov10 like RNA helicase 1 | 22 | 22q13.33 | 605794 | 54456 |
| MPZL2 | myelin protein zero like 2 | 11 | 11q23.3 | 604873 | 10205 |
| MSH4 | mutS homolog 4 | 1 | 1p31.1 | 602105 | 4438 |
| MSH5 | mutS homolog 5 | 6 | 6p21.33 | 603382 | 4439 |
| NANOS3 | nanos C2HC-type zinc finger 3 | 19 | 19p13.12 | 608229 | 342977 |
| NR5A1 | nuclear receptor subfamily 5 group A member 1 | 9 | 9q33.3 | 184757 | 2516 |
| NSUN7 | NOP2/Sun RNA methyltransferase family member 7 | 4 | 4p14 | 617185 | 79730 |
| OR2W3 | olfactory receptor family 2 subfamily W member 3 | 1 | 1q44 | 616729 | 343171 |
| P2RX1 | purinergic receptor P2X 1 | 17 | 17p13.2 | 600845 | 5023 |
| PDHA2 | pyruvate dehydrogenase E1 subunit alpha 2 | 4 | 4q22.3 | 179061 | 5161 |
| PFN4 | profilin family member 4 | 2 | 2p23.3 | 620046 | 375189 |
| PGAP1 | post-GPI attachment to proteins inositol deacylase 1 | 2 | 2q33.1 | 611655 | 80055 |
| PICK1 | protein interacting with PRKCA 1 | 22 | 22q13.1 | 605926 | 9463 |
| PIWIL1 | piwi like RNA-mediated gene silencing 1 | 12 | 12q24.33 | 605571 | 9271 |
| PLCZ1 | phospholipase C zeta 1 | 12 | 12p12.3 | 608075 | 89869 |
| PMFBP1 | polyamine modulated factor 1 binding protein 1 | 16 | 16q22.2 | 618085 | 83449 |
| PNLDC1 | PARN like ribonuclease domain containing exonuclease 1 | 6 | 6q25.3 | 619529 | 154197 |
| POLG | DNA polymerase gamma, catalytic subunit | 15 | 15q26.1 | 174763 | 5428 |
| POLL | DNA polymerase lambda | 10 | 10q24.32 | 606343 | 27343 |
| PRDM9 | PR/SET domain 9 | 5 | 5p14.2 | 609760 | 56979 |
| PRM1 | protamine 1 | 16 | 16p13.13 | 182880 | 5619 |
| PRND | prion like protein doppel | 20 | 20p13 | 604263 | 23627 |
| PTPN11 | protein tyrosine phosphatase non-receptor type 11 | 12 | 12q24.13 | 176876 | 5781 |
| QRICH2 | glutamine rich 2 | 17 | 17q25.1 | 618304 | 84074 |
| RABL2A | RAB, member of RAS oncogene family like 2A | 2 | 2q14.1 | 605412 | 11159 |
| RNF212 | ring finger protein 212 | 4 | 4p16.3 | 612041 | 285498 |
| RPL10L | ribosomal protein L10 like | 14 | 14q21.2 | 619655 | 140801 |
| SECISBP2 | SECIS binding protein 2 | 9 | 9q22.2 | 607693 | 79048 |
| SEPTIN12 | septin 12 | 16 | 16p13.3 | 611562 | 124404 |
| SHOC1 | shortage in chiasmata 1 | 9 | 9q31.3 | 618038 | 158401 |
| SLC26A8 | solute carrier family 26 member 8 | 6 | 6p21.31 | 608480 | 116369 |
| SNRPA1 | small nuclear ribonucleoprotein polypeptide A' | 15 | 15q26.3 | 603521 | 6627 |
| SOHLH1 | spermatogenesis and oogenesis specific basic helix-loop-helix 1 | 9 | 9q34.3 | 610224 | 402381 |
| SOX8 | SRY-box transcription factor 8 | 16 | 16p13.3 | 605923 | 30812 |
| SPACA1 | sperm acrosome associated 1 | 6 | 6q15 | 612739 | 81833 |
| SPAG6 | sperm associated antigen 6 | 10 | 10p12.2 | 605730 | 9576 |
| SPAG17 | sperm associated antigen 17 | 1 | 1p12 | 616554 | 200162 |
| SPATA16 | spermatogenesis associated 16 | 3 | 3q26.31 | 609856 | 83893 |
| SPEF2 | sperm flagellar 2 | 5 | 5p13.2 | 610172 | 79925 |
| SPEM1 | spermatid maturation 1 | 17 | 17p13.1 | 615116 | 374768 |
| SPOCD1 | SPOC domain containing 1 | 1 | 1p35.2 | 619038 | 90853 |
| SSX1 | SSX family member 1 | X | Xp11.23 | 312820 | 6756 |
| STAG3 | STAG3 cohesin complex component | 7 | 7q22.1 | 608489 | 10734 |
| STK11 | serine/threonine kinase 11 | 19 | 19p13.3 | 602216 | 6794 |
| STRC | stereocilin | 15 | 15q15.3 | 606440 | 161497 |
| SUN5 | Sad1 and UNC84 domain containing 5 | 20 | 20q11.21 | 613942 | 140732 |
| SYCP2 | synaptonemal complex protein 2 | 20 | 20q13.33 | 604105 | 10388 |
| TBC1D20 | TBC1 domain family member 20 | 20 | 20p13 | 611663 | 128637 |
| TBC1D21 | TBC1 domain family member 21 | 15 | 15q24.1 | 620387 | 161514 |
| TBC1D25 | TBC1 domain family member 25 | X | Xp11.23 | 311240 | 4943 |
| TDRD9 | tudor domain containing 9 | 14 | 14q32.33 | 617963 | 122402 |
| TEKT2 | tektin 2 | 1 | 1p34.3 | 608953 | 27285 |
| TEKT3 | tektin 3 | 17 | 17p12 | 612683 | 64518 |
| TERB1 | telomere repeat binding bouquet formation protein 1 | 16 | 16q22.1 | 617332 | 283847 |
| TERB2 | telomere repeat binding bouquet formation protein 2 | 15 | 15q21.1 | 617131 | 145645 |
| TEX11 | testis expressed 11 | X | Xq13.1 | 300311 | 56159 |
| TEX14 | testis expressed 14, intercellular bridge forming factor | 17 | 17q22 | 605792 | 56155 |
| TGFB1 | transforming growth factor beta 1 | 19 | 19q13.2 | 190180 | 7040 |
| TTC21A | tetratricopeptide repeat domain 21A | 3 | 3p22.2 | 611430 | 199223 |
| TTC29 | tetratricopeptide repeat domain 29 | 4 | 4q31.22 | 618735 | 83894 |
| UBE2B | ubiquitin conjugating enzyme E2 B | 5 | 5q31.1 | 179095 | 7320 |
| USP9Y | ubiquitin specific peptidase 9 Y-linked | Y | Yq11.221 | 400005 | 8287 |
| USP26 | ubiquitin specific peptidase 26 | X | Xq26.2 | 300309 | 83844 |
| UTP14C | UTP14C small subunit processome component | 13 | 13q14.3 | 608969 | 9724 |
